# The Oxytocic Action of Quinine

**Published:** 1884-02-20

**Authors:** 


**The Oxytocic Action of Quinine.**—Mr. Hartigan, M. K. Q. C. P., of Hong Kong, writes:

"In three different cases I have had on several occasions to discontinue the use of quinine because it brought on "labor-pains," though the doses used were small, varying from three to five grains. In one of these, during the previous pregnancy, another medical man used quinine, and discontinued it for a similar reason. All three were in fair general health, suffering only from slight malarious fever, and had never aborted. One case has come under my notice in which abortion took place, without apparent cause, after a ten grain dose of quinine. The patient was the mother of several children, had not previously aborted, was in good health, and took the quinine to cure facial neuralgia. I know of another case of abortion occurring under similar circumstances after quinine. This action of the drug is known to the Chinese, who take it (I am told, with success) for the purpose of producing abortion, following its use by copious draughts of hot tea. I have myself heard a Chinese "amah," (*i. e.*, female servant), recommend it. Quinine certainly, in some cases, increases the menstrual flow."—*British Med. Journal.*

**Application for Warts.**—Dr. Cordes, of Geneva, states (Journal de Therapeutique) that he has always found the following application successful : Iodine six, crystallized carbolic acid twenty-one, and alcohol two parts and a half by weight. After scraping the wart or cutting it down to a level with the skin (without causing it to bleed), he touches the wart with a few drops of the above solution. In a minute it becomes soft, and allows of another scraping and a new application ; and sometimes even a third scraping and application can be made without causing bleeding. —*Med. Times and Gaz.*

